# Tau mediates microtubule bundle architectures mimicking fascicles of microtubules found in the axon initial segment

**DOI:** 10.1038/ncomms12278

**Published:** 2016-07-25

**Authors:** Peter J. Chung, Chaeyeon Song, Joanna Deek, Herbert P. Miller, Youli Li, Myung Chul Choi, Leslie Wilson, Stuart C. Feinstein, Cyrus R. Safinya

**Affiliations:** 1 Materials Department, Physics Department, Molecular, Cellular, and Developmental Biology Department, University of California, Santa Barbara, California 93106, USA; 2 Chemistry and Biochemistry Department, University of California, Santa Barbara, California 93106, USA; 3 Neuroscience Research Institute and Molecular, Cellular, and Developmental Biology Department, University of California, Santa Barbara, California 93106, USA; 4 Materials Research Laboratory, University of California, Santa Barbara, California 93106, USA; 5 Department of Bio and Brain Engineering, Korea Advanced Institute of Science and Technology (KAIST), Daejeon 34141, Korea

## Abstract

Tau, an intrinsically disordered protein confined to neuronal axons, binds to and regulates microtubule dynamics. Although there have been observations of string-like microtubule fascicles in the axon initial segment (AIS) and hexagonal bundles in neurite-like processes in non-neuronal cells overexpressing Tau, cell-free reconstitutions have not replicated either geometry. Here we map out the energy landscape of Tau-mediated, GTP-dependent ‘active' microtubule bundles at 37 °C, as revealed by synchrotron SAXS and TEM. Widely spaced bundles (wall-to-wall distance *D*_w–w_≈25–41 nm) with hexagonal and string-like symmetry are observed, the latter mimicking bundles found in the AIS. A second energy minimum (*D*_w–w_≈16–23 nm) is revealed under osmotic pressure. The wide spacing results from a balance between repulsive forces, due to Tau's projection domain (PD), and a stabilizing sum of transient sub-*k*_B_*T* cationic/anionic charge–charge attractions mediated by weakly penetrating opposing PDs. This landscape would be significantly affected by charge-altering modifications of Tau associated with neurodegeneration.

## Abstract (web summary)

Tau, an intrinsically disordered axonal protein, binds to and regulates microtubule dynamics. Here, the authors use SAXS and electron microscopy to examine the architectures of microtubule bundles, including those mimicking microtubule fascicles in the axon initial segment.

## Graphical abstract

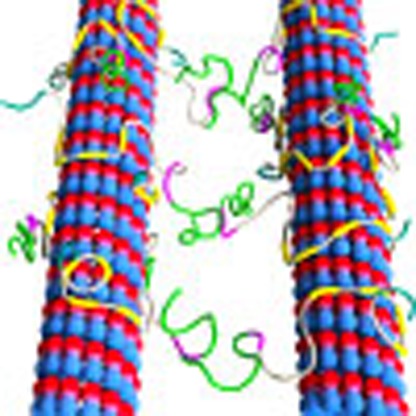

Microtubules, a component of the eukaryotic cytoskeleton, are made up of αβ-tubulin heterodimers that dynamically assemble into hollow nanotubes composed of straight protofilaments^1^. Microtubules are involved in a wide variety of cell functions (for example, intracellular trafficking, cell motility and chromosome segregation) through functionalization, in part, by microtubule-associated proteins (MAPs). One MAP in particular, Tau, is localized to neuronal axons and stabilizes microtubules upon binding (Fig. 1a), by partially suppressing microtubule dynamic instability (alternating periods of polymerization of tubulin into microtubules interrupted by catastrophe, or the rapid disassembly of microtubules following loss of the microtubule GTP cap). While Tau is developmentally regulated in neurons, Tau dysfunction in mature axons has been linked to many neurodegenerative ‘tauopathies' (including Alzheimer's^2^, FTDP-17 (ref. 3), and, more recently, chronic traumatic encephalopathy in athletes suffering concussions^4^). Tau consists of an amino-terminal tail (NTT) containing a projection domain (PD) and proline-rich region, followed by the microtubule-binding region (MTBR) and a carboxyl-terminal tail (CTT). Alternative splicing results in six wild-type (WT) isoforms (Fig. 1b); the MTBR either has four or three imperfect repeats (4R- or 3R-Tau isoforms), depending on the expression/non-expression of exon 10. The PD length depends on the expression of exons 2 and/or 3 with +/+, +/− and −/−, resulting in long (-L), medium (-M) and short (-S) length PDs, respectively.

While it is generally accepted that Tau interacts with individual microtubules, the interactions between microtubules mediated by Tau remains unclear. Before the discovery of Tau, groundbreaking electron microscopy studies of the fine structure of axons in mature rat hippocampal neurons revealed^5^,^6^ that the axon initial segment (AIS) contained widely spaced (*D*_w–w_≈25–30 nm) string-like microtubule bundles (‘fascicles of microtubules', Fig. 1c). Fascicles are now believed^7^ to be a cardinal feature of the AIS, acting as a filter by allowing passage of kinesin motors with axon-specific cargo and thus contributing to neuronal polarity (that is, ensuring distinct molecular environments for axons and dendrites). Later, seminal studies^8^,^9^ of non-neuronal cells overexpressing transfected Tau cDNA had concluded that the Tau PD determines inter-microtubule distances in observed widely spaced hexagonally ordered microtubule arrays (Fig. 1d), although the studies could not discern whether bundles result from the presence of Tau-mediated attractions or due to a repulsive lattice under confinement. The answer to the very important question of whether Tau, which is localized to the axon region, induces microtubule bundles similar to those found in the AIS remains controversial. Cell-free reconstitutions^10^,^11^ resulted in no microtubule bundles, which led to the conclusion that the absence of bundles was due to the purely repulsive nature of Tau. Complicating matters was the frequent use of paclitaxel to stabilize microtubules^12^,^13^. Synchrotron small-angle X-ray scattering (SAXS) has shown^14^ that WT Tau does not bundle paclitaxel-stabilized microtubules over the relevant Tau-coverage regime in axons^15^. In eliminating microtubule dynamic instability, it was possible that the normal behaviour of microtubules was being suppressed, without it being clear that paclitaxel-stabilized results were reflective of biological reality.

The current study was designed to elucidate the molecular mechanism of Tau-mediated microtubule–microtubule interactions in paclitaxel-free reaction mixtures of tubulin, GTP and WT Tau/truncated Tau (at Tau to tubulin–dimer molar ratios, Φ_Tau_=1/40 to 1/5) under dissipative out-of-equilibrium conditions at 37 °C (that is, mimicking the cytoskeletal environment of nerve cells with samples consuming the energy released by GTP hydrolysis). Synchrotron SAXS and plastic-embedded transmission electron microscopy (TEM) were used to obtain both angstrom-resolution ensemble-averaged structural information and nanometer-scale real-space local fine structure, respectively.

Our study reveals steady-state structures that are stable over time (>24 h). Synchrotron SAXS studies under osmotic pressure allows us to map out the energy landscape of Tau-mediated, GTP-dependent active microtubule bundles at 37 °C. In the absence of applied osmotic pressure, the microtubule reaction mixture exhibits Tau-induced phase separation into microtubule bundles (forming domains of high and low concentrations of microtubules in optical microscopy), unambiguously demonstrating the presence of an attractive component to Tau-mediated interactions between microtubules, as opposed to previous studies that had concluded that Tau acted as a purely repulsive spacer^10^,^16^. SAXS and TEM reveal widely spaced bundles (energy minimum at microtubule wall-to-wall distance *D*_w–w_≈25–41 nm) with hexagonal and string-like symmetry, the latter mimicking bundles found in the AIS. We propose a mechanism in which aggregate sub-*k*_B_*T* cationic/anionic charge–charge attractions by weakly penetrating Tau PDs on opposing microtubules stabilize active bundles. This mechanism is contingent on the overall microtubule length and thereby reconciles the lack of microtubule bundles in previous cell-free reconstitutions^10^,^11^,^14^. With applied osmotic pressure, a second minimum (*D*_w–w_≈16–23 nm), indicative of antiparallel dipole–dipole interactions of interpenetrating Tau PDs, is revealed. Notably, we find that contrary to current dogma, the CTT alone is able to mediate relatively wide spacings in the absence of the NTT.

## Results

### SAXS reveals Tau-mediated hexagonally ordered MT bundles

**Figure 1:**
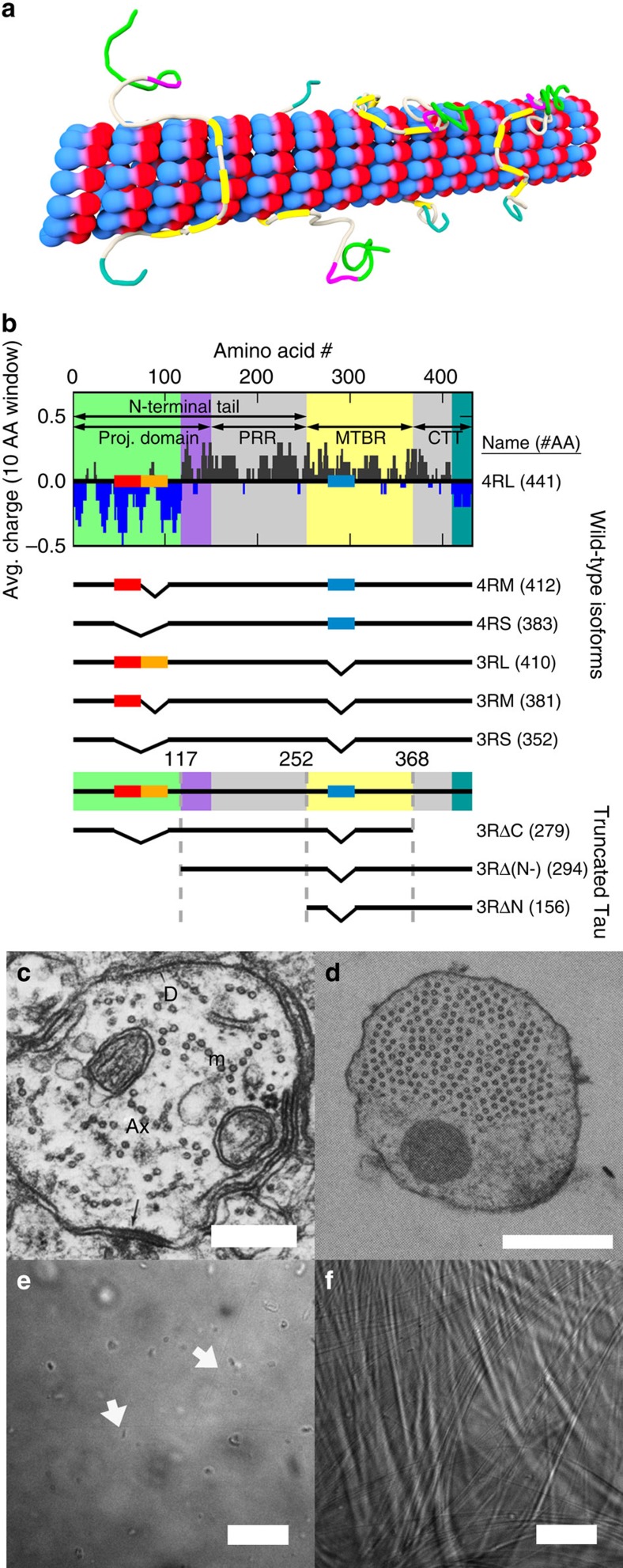
Tau-mediated microtubule assemblies and Tau charge distribution. (**a**) Cartoon showing Tau binding to a microtubule (red/blue) through its microtubule-binding region (yellow), with the projection domain (green/purple) and CTT (grey/teal) extending off the microtubule surface. (**b**) The average charge (dark blue for anionic character and dark grey for cationic character) of fully expressed 4RL Tau (top) as a function of primary sequence, with alternative splicing of exons 2 (red rectangle), 3 (orange) and 10 (blue) resulting in the five additional wild-type isoforms. Wild-type Tau consists of the amino-terminal tail (NTT), which includes the projection domain (PD, green/purple background) and proline-rich region (PRR, grey), followed by the microtubule-binding region (MTBR, yellow) and carboxyl-terminal tail (CTT, grey/teal). Truncated Tau constructs were designed to understand the domain dependence of the CTT (3RSΔC, missing CTT), anionic component of the projection domain (3RΔ(N-), missing anionic block of NTT) and the entire NTT (3RΔN, missing NTT). (**c**) Prior electron microscopy revealed linear microtubule bundles in the axon initial segment (adapted from Peters *et al.*^5^ and reprinted by permission of Oxford University Press, USA). (**d**) Subsequent Tau cDNA transfection of Sf9 cells revealed hexagonal arrays of microtubules in neurite-like processes (adapted from Chen *et al.*^8^ and reprinted by permission from Macmillan Publishers Ltd: Nature **360,** 674–677, ©1992). (**e**) While individual microtubules (arrows) can be identified via differential interference contrast (DIC) microscopy, no discernible bundles form in paclitaxel-free microtubules without Tau. (**f**) With centrifugation (9,500*g* for 30 min at 37 °C), microtubules form clear and stable bundles when assembled with Tau (Φ_3RL_=1/20), demonstrating an attractive component of the Tau-mediated microtubule–microtubule interaction. Scale bars, 250 nm (**c**); 500 nm (**d**); 20 μm (**e**–**f**).

**Figure 2:**
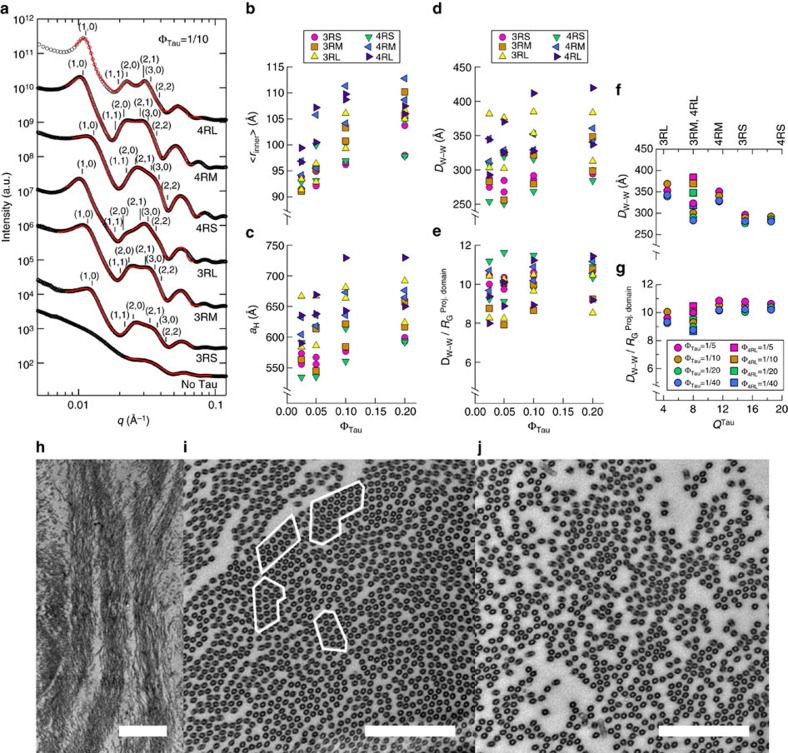
SAXS and TEM show that Tau-assembled microtubules in active bundles recapitulate key *in vivo* features of microtubule spacing and linear bundles. (**a**) Azimuthally averaged SAXS data of WT Tau and microtubules registers Bragg peak positions consistent with hexagonal lattices for all six isoforms, as opposed to just microtubule form factor for no Tau (bottom profile). (**b**–**e**) Line-shape analysis of the SAXS data (resultant fits in red in **a**) yields the ensemble-averaged microtubule inner radius <*r*_in_> (**b**), hexagonal lattice parameter *a*_H_ (**c**), wall-to-wall distance *D*_w–w_ (**d**) and *D*_w–w_ normalized by the calculated projection domain radius of gyration, *R*_G_^PD^ (**e**; see Methods). Parameters plotted are the result of line-shape analyses of two representative data measurements after 12 h to ensure equilibration, with samples made from independent tubulin purifications and Tau expressions/purifications. (**f**,**g**) The average *D*_w–w_ and *D*_w–w_/*R*_G_^PD^ as a function of Tau net charge (*Q*^Tau^) shows a monotonic decrease in *D*_w–w_ and a nearly constant *D*_w–w_/*R*_G_^PD^≈8–11, respectively. (3RM and 4RL, coincidentally, have the same charge by AA sequence and thus 4RL has been specially labelled.) (**h**) Electron microscopy of microtubules assembled with Tau (Φ_3RM_=1/20) at low magnification show distinct bundled domains, demonstrating phase separation. (**i**) Domains of hexagonally ordered arrays of microtubules (identified in white outlines, Φ_3RL_=1/20) with vacancies likely resulting from the suppressed (but still occurring) dynamic instability. (**j**) Linear bundles of microtubules (Φ_3RL_=1/20), a result of extensive vacancy introduction and mimicking string-like microtubule bundles in the AIS. In **i** and **j**, the staining process exaggerates the microtubule wall thickness. Scale bars, 1 μm (**h**); 500 nm (**i**,**j**).

SAXS is especially well suited to investigate Tau-directed higher-order assembly of microtubules (as seen at low resolution via differential interference contrast (DIC) microscopy; Fig. 1e,f), as solution scattering yields assembly structures in near-physiological conditions without tags/labels. Azimuthally averaged scattering of microtubules co-assembled with WT Tau isoforms (Φ_Tau_=1/10) registers Bragg peak positions consistent with a two-dimensional (2D) hexagonal array (*q*_10_, *q*_11_=3^1/2^*q*_10_, *q*_20_=2*q*_10_, *q*_21_=7^1/2^*q*_10_, *q*_30_=3*q*_10_, *q*_22_=12^1/2^*q*_10_) of microtubules with centre-to-centre distance *a*_H_=4π/(3^1/2^*q*_10_) (Fig. 2a). Some peak positions are not apparent due to their proximity to the form factor minima (in particular, *q*_11_ and *q*_22_), necessitating line-shape analysis to separate scattering from individual microtubules (form factor, see Fig. 2a, bottom profile), the lattice of microtubules (structure factor) and background small-angle scattering. Following previous work^14^,^17^, we modelled microtubules as hollow cylinders with ensemble-averaged inner radius <*r*_in_> (a fit parameter) and constant wall thickness *δ* (49 Å, in agreement with electron microscopy of microtubules^18^). Each structure factor peak at reciprocal lattice vector *q*_*hk*_=*q*_10_(*h*^2^+*k*^2^+*hk*)^1/2^ was represented as squared lorentzians (fit results as red solid lines in Fig. 2a, see Methods).

Even after ≈24 h, there are no major changes in scattering (Supplementary Fig. 1), indicating that although the microtubules are dynamic (that is, hydrolyzing GTP), they have reached a steady state. The fit parameters, <*r*_in_> and *a*_H_, calculated microtubule wall-to-wall distance *D*_w–w_ (=*a*_H_−2(<*r*_in_>+*δ*)) and *D*_w–w_ normalized to the PD radius of gyration (*R*_G_^PD^) in solution (2.8/3.3/3.8 nm for -S/-M/-L Tau isoforms, see Methods) are plotted in Fig. 2b–e, as a function of increasing Φ_Tau_. The *D*_w–w_≈25–41 nm is similar to inter-microtubule spacings seen in cells (Fig. 1c). For both three-repeat and four-repeat Tau isoforms, increasing PD length (increasing anionic block size, Fig. 1b) leads to increases in *D*_w–w_ (for example, at Φ_Tau_=1/10 in Fig. 2d *D*_w–w_ increases from ≈28–29 nm (26–31 nm) to 35–38 nm (33–41 nm) in going from 3RS (4RS) to 3RL (4RL)). This behaviour is consistent with observations in transfected cells^8^,^9^, where Tau isoforms with longer PDs exhibit microtubule bundles with larger spacing. Notably, *D*_w–w_/*R*_G_^PD^ is nearly constant (≈8–11, Fig. 2e). By plotting *D*_w–w_ and *D*_w–w_/*R*_G_^PD^ for the six WT isoforms as a function of the total overall charge of Tau (*Q*^Tau^, Fig. 2f,g), we see that *D*_w–w_ decreases systematically as the overall cationic charge of Tau increases. We note that the decrease in *D*_w–w_ with increasing charge is accompanied by the simultaneous decrease in the size of the PD (that is, increases in Tau charge correspond to anionic inserts being removed). Thus, both the Tau PD size and electrostatics of Tau plays a role in mediating these widely spaced bundles.

### TEM reveals active microtubule architecture mimicking that of the AIS

TEM independently confirms phase separation of microtubules into bundles (Fig. 2h, Φ_3RM_=1/20) seen in DIC microscopy (Fig. 1f). TEM cross-sections exhibit microtubule domains in widely spaced hexagonally ordered 2D arrays (Φ_3RL_=1/20, white outlines in Fig. 2i), consistent with SAXS diffraction peaks. However, there is also an additional feature not apparent in the SAXS data: vacancies amidst areas of high microtubule density (akin to vacancy defects in crystalline materials). The vacancies are likely the result of the dissipative nature of the reaction mixture: although Tau partially suppresses dynamic instability in a Tau concentration-dependent manner^19^, microtubule dynamic instability is still occurring (for Φ_Tau_≤1/5) and introduces significant vacancies in the microtubule array. While the more ordered regions are readily picked up in SAXS, TEM also reveals significantly less-ordered regions, where microtubules appear to form linear bundles (Φ_3RL_=1/20, Fig. 2j), mimicking string-like bundles (‘fascicles of microtubules'^6^) found in the AIS (Fig. 1c). The occurrence of these linear bundles in regions of low microtubule concentration may be the result of simultaneous attractive and repulsive interactions of comparable strength between Tau-coated microtubules at wide spacings.

### Truncated Tau shows Tau PD is unnecessary for microtubule bundles

**Figure 3:**
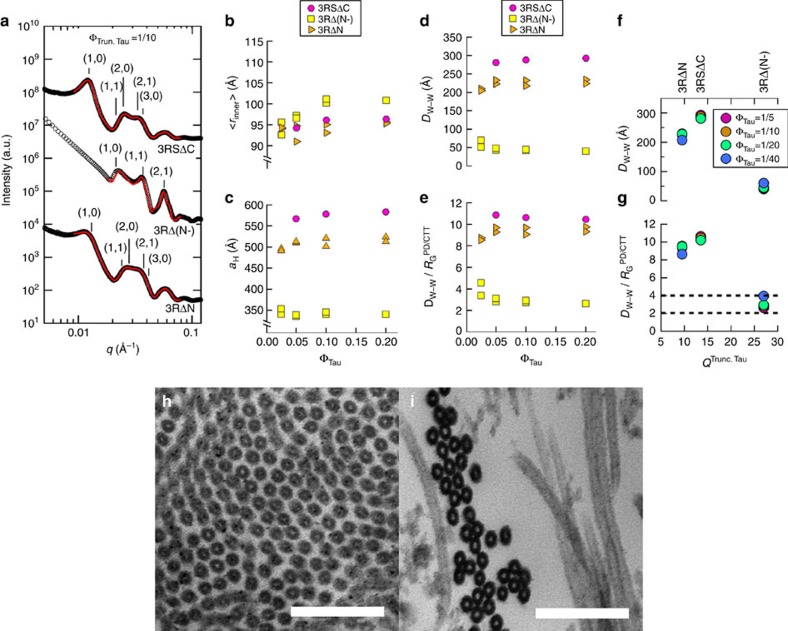
SAXS and TEM of microtubules assembled with truncated Tau show widely and tightly spaced microtubule bundles. (**a**) Azimuthally averaged SAXS data of truncated Tau exhibits scattering consistent with widely spaced (3RSΔC (missing CTT, top line-shape, *q*_10_=0.0126 Å^−1^) and 3RΔN (missing NTT, bottom line shape, *q*_10_=0.0139 Å^−1^)) and tightly spaced [3RΔ(N-), (missing anionic section of PD, middle line shape, *q*_10_=0.0213 Å^−1^)) hexagonally ordered microtubules. (**b**–**e**) Line-shape analysis of the SAXS data (resultant fits in red in **a**) yields <*r*_in_> (**b**), *a*_H_ (**c**), *D*_w–w_ (**d**) and *D*_w–w_ normalized by *R*_G_ of the PD (3RSΔC), remaining PD (3RΔ(N-)), or CTT (3RΔN) (**e**). Parameters plotted are the result of line-shape analyses of two representative data measurements after 12 h to ensure equilibration, with samples made from independent tubulin purifications and Tau expressions/purifications. For the 3RSΔC sample, one representative data measurement are plotted. (**f**,**g**) The average *D*_w–w_ and *D*_w–w_/*R*_G_^PD^ as a function of truncated Tau net charge (*Q*^Trun. Tau^) reveals a disparity in the data for 3RΔ(N-), when compared with WT Tau (Fig. 2f,g), indicative of a different interaction regime between microtubules, likely induced by correlated density fluctuations. (**h**) TEM of widely spaced microtubule bundles (Φ_3RΔN_=1/20) despite a lack of an entire NTT (and thus, lacking the projection domain). (**i**) Closely packed microtubules (Φ_3RΔ(N-)_=1/20) upon elimination of the anionic component of the projection domain suggesting an interaction mediated by correlated density fluctuations of the overall cationic 3RΔ(N-) on the microtubule surface. Scale bars, 150 nm (**h**,**i**).

To further elucidate the nature of Tau-mediated microtubule–microtubule interactions, truncated Tau mutants (Fig. 1a, truncated Tau) were expressed/purified, and used for similar SAXS measurements (Fig. 3a) and subsequent parameter extraction from line-shape analysis (Fig. 3b–g): 3RSΔC (truncation of the entire CTT), 3RΔ(N-) (truncation of the anionic component of the NTT) and 3RΔN (truncation of the entire NTT). CTT elimination of 3RS Tau (3RSΔC) has scattering associated with widely spaced bundles (Fig. 3a, top profile) and extracted parameters <*r*_in_>, *a*_H_ and *D*_w–w_ (Fig. 3b–d) similar to that of 3RS WT Tau (Fig. 2b–d), strongly indicating that the CTT is not critical to the WT mechanism of widely spaced bundles. Elimination of the entire anionic block from the PD (3RΔ(N-)) collapses the bundles (Fig. 3a, middle profile, Fig. 3d, *D*_w–w_≈4–5 nm), with a tight microtubule wall-to-wall spacing comparable to the radius of gyration of 3RΔ(N-) (*R*_G_=4.79 nm, see Methods)^20^. This result indicates that the anionic block of the PD (a charged polymer containing overall anionic and cationic blocks) presents the dominant component of the repulsive barrier preventing neighbouring microtubules from getting closer.

Removal of the entire NTT of Tau (3RΔN) results in a highly unexpected finding, where the SAXS data (Fig. 3a, bottom profile) give *D*_w–w_≈22–24 nm (Fig. 3d) compatible with widely spaced bundles, despite the nominal size (*R*_G_=4.0 nm) of 3RΔN. The relatively wide spacing seen upon elimination of the NTT (3RΔN) indicates that the NTT is unnecessary for widely spaced bundling (under these conditions) and that, in its absence, the CTT (Fig. 1a) plays an equivalent role in determining the inter-microtubule interactions. TEM of microtubules assembled with 3RΔN and 3RΔ(N-) (Fig. 3h,i) clearly shows microtubule bundles with relatively wide inter-microtubule spacing and in close contact, respectively, consistent with the SAXS data. For 3RΔN and 3RSΔC, *D*_w–w_/*R*_G_ (≈8–11, Fig. 3e,g) is consistent with the WT ratio (Fig. 2e,g), suggesting a similar mechanism of inter-microtubule interactions. However, 3RΔ(N-) presented markedly smaller *D*_w–w_ (≈5 nm) and *D*_w–w_/*R*_G_ (≈2–4, Fig. 3e,g), suggesting a different inter-microtubule interaction regime.

### Second energy minimum of bundles accessed via osmotic stress

**Figure 4:**
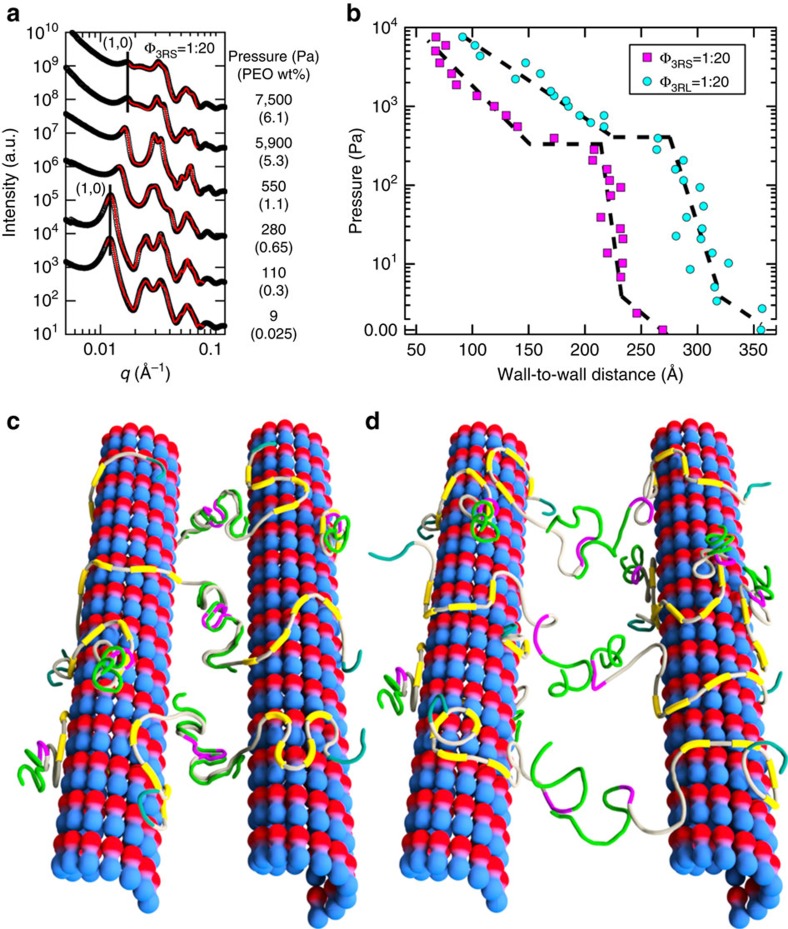
Direct force measurements of Tau-mediated active microtubule bundles reveal distinct energy minima. (**a**) Representative azimuthally averaged SAXS data for Φ_3RS_=1/20 at selected increasing pressures resulting in increasing peak position of *q*_10_, reflecting the decrease in the hexagonal lattice parameter *a*_H_. (**b**) The measured wall-to-wall distances (*D*_w–w_) for 3RS- and 3RL-mediated microtubule bundles show a sudden transition (≈5 nm decrease) at applied osmotic pressure *P*≈300–400 Pa, indicative of a secondary energy minimum for microtubule bundles. (**c**) A cartoon of the osmotic-pressure-induced secondary minimum at intermediate *D*_w–w_ with antiparallel dimerization occurring between the anionic section of the NTT (green) and the cationic part of the PD (purple) plus cationic proline-rich region (grey). (**d**) A cartoon of the widely spaced energy minimum, with Tau-mediating microtubule bundles by transient charge–charge attractions between the cationic residues (purple/grey) and the anionic residues (green) in the amino-terminal tail.

To understand the molecular mechanism of the Tau-mediated interactions between microtubules in active bundles, the force response behaviour of bundles was measured via SAXS of reaction mixtures under osmotic stress. We used high molecular-weight PEO-100k (size≈40 nm, see Methods) to ensure that polymer depletant did not penetrate the inter-microtubule region in the widely spaced bundles, creating a polymer concentration exterior to microtubule bundles and thus exerting an osmotic pressure (*P*) on the bundle itself. By this method (Fig. 4a), we measured the microtubule wall-to-wall spacing *D*_w–w_ of bundles induced by 3RS and 3RL WT isoforms as a function of increasing *P* (Fig. 4b). The *P*–*D*_w–w_ curves for both WT isoforms exhibit an initial soft repulsion with *D*_w–w_ decreasing ≈3–4 nm up to *P*≈40 Pa followed by a steep increase in slope (with *D*_w–w_ decreasing ≈2–3 nm for 40 Pa<*P*<≈300–400 Pa) consistent with a highly repulsive barrier due to the PD with anionic blocks, resisting osmotic compression. Remarkably, above a critical pressure *P*_c_ (≈300 and 400 Pa for 3RS and 3RL, respectively), there is an abrupt ≈5 nm decrease in *D*_w–w_ from ≈21.5 to ≈16.5 nm for 3RS and ≈27.5 to≈22.5 nm for 3RL. This sudden jump is reflected in the SAXS data as a sudden large shift in peak position as *P* is increased to just above *P*_c_ (lines in Fig. 4a are a guide to the first peak below and above *P*_c_).

## Discussion

The osmotic pressure data and, more specifically, the abrupt transition above *P*_c_ are consistent with the onset of antiparallel dimerization between fully interpenetrating dipolar Tau PDs on opposing microtubule surfaces (Fig. 4c). Microtubule bundles at this intermediate spacing are in a second local energy minimum distinct from widely spaced bundles in the absence of PEO. Several findings support this model. First, reversibility measurements show this local minimum is stable, as bundles for *P*>*P*_c_ do not relax to their previous spacings upon removal of PEO-100k but instead relax to the *D*_w–w_ associated with *P*_c_. This implies the barrier between the second and widely spaced minima is greater than thermal energy *k*_B_*T*. Second, the wall-to-wall spacing observed for this local minimum for intermediate spacing bundles (*D*_w–w_≈22.5 nm for 3RL) is consistent with the recent work showing that PDs for microtubule-bound Tau are in an extended conformation (size≈23 nm for the -L isoforms), twice that of the Tau PD physical diameter in solution^21^.

Considering the minimum associated with widely spaced bundles, several findings with WT Tau isoforms point to the repulsive component emanating from the PD containing the anionic block: the steep repulsive barrier when PD chains are pushed together under osmotic pressure and the increase in *D*_w–w_ with increasing PD length (and increasing anionic block size, Fig. 2d). The observation of microtubule phase separation into widely spaced bundles (Figs 1f and 2h), upon addition of small amounts of WT Tau (as low as Φ_Tau_=1/40), demonstrates a Tau-mediated attractive component of the energy minimum, which overcomes the repulsive component, stabilizing microtubule bundles against dilution. To reconcile these wide spacings, we note that it was recently discovered^20^ that the effective size of the PD was more extended on the microtubule surface (≈23 nm for -L isoforms) than SAXS solution data would indicate. This effective size, when put in context of the large *D*_w–w_ (≈35–38 nm for 3RL), strongly supports a model where only PD end regions interact with each other to create an attraction potential. Although the Tau PD is overall anionic, locally it is a polyampholyte containing positive and negative residues. In addition, the short Debye screening length (≈9.8 Å) of the reaction mixture solution and consistently wide spacings (relative to PD *R*_G_ in solution, see Fig. 2f) strongly suggest a mechanism mediated by short-ranged attractions between the ends of opposing and extended Tau PDs for all WT isoforms.

Accordingly, we propose that the attraction is provided by transient short-range charge–charge attractions between cationic/anionic residues of weakly penetrating Tau PDs near the midplane-layer between opposing microtubule surfaces (Fig. 4d). The proposed short-range attraction would still require transient extensions beyond the average length. Indeed, the SAXS measurements are ensemble-averaged over a finite time, in which transient, extended conformations are possible, as seen in Monte-Carlo simulations of the PD of 3RS Tau on microtubule surfaces^22^. While individually these attractions are weak^23^, the sum of these interactions over the entire microtubule length should be sufficient to stabilize microtubule bundles against thermal fluctuations. (This phenomenon of collective action mediated by a sum of weak attractions is a recurring theme in intrinsically disordered systems, as seen in the nuclear pore complex^24^,^25^ and neurofilament sidearms^26^,^27^,^28^.) For Tau-stabilized microtubules (microtubule length≈20 μm)^11^ in hexagonally ordered active bundles, an energy depth of 10–20 *k*_B_*T* between microtubules would require ≈0.05–0.1 *k*_B_*T* per Tau–Tau interaction at Φ_tau_=1/40. This mechanism is consistent with shorter microtubules not bundling^14^. Indeed, cell-free constitutions in which microtubule bundling was not observed often involved shorter, paclitaxel-stabilized microtubules^10^,^14^ (microtubule length ≈2 μm)^29^.

A model of polyampholyte-mediated attraction near the midplane-layer dovetails with the truncated Tau data: upon deletion of the entire NTT (3RΔN), the CTT plays an analogous role. The CTT, much like the NTT, has dipole-like characteristics, with a cationic block and anionic block distal to the Tau MTBR (Fig. 1b). Thus, like the NTT, we expect the CTT to be similarly extended off the microtubule surface, as underscored by comparing *D*_w–w_/*R*_G_ for WT Tau (≈8–11, normalized by the PD *R*_G_ in solution, Fig. 2f,g) and 3RΔN (≈8–10, normalized by the CTT *R*_G_ in solution, Fig. 3f,g). However, elimination of the PD anionic block (3RΔ(N-)) results in an overall highly cationic protein, which results in a collapse in spacing between microtubule bundles. This collapse may be due to a similar phenomenon where counterions adopt a non-uniform distribution around charged objects, inducing an attraction via correlated counterion density fluctuations^30^ (that is, similar to the van der Waals attraction mediated by correlated, transient dipoles). This phenomenon was observed in tightly spaced microtubule bundles induced by smaller polyamines such as spermine (4+)^17^. Remarkably, this ion correlation mechanism predicts that *D*_w–w_≈physical size^31^ of the macromolecular counterion (=2(5/3)^1/2^*R*_G_), consistent with *D*_w–w_/*R*_G_≈2–4 for 3RΔ(N-) (Fig. 3e,g).

The proposed mode by which Tau bundles microtubules may have major implications for post-translational modifications of Tau associated with neurodegeneration, especially phosphorylation. In tauopathies, both cytosolic Tau and aggregated Tau in neurofibrillary tangles are hyperphosphorylated^32^,^33^. Our mechanism predicts that increasing Tau phosphorylation would suppress Tau-mediated attractions and bundle formation. Although the identity of MAPs involved in fascicle formation in the AIS is not fully known, our results suggest that hyperphosphorylation of MAPs in the AIS (for example, either MAP Tau or other MAPs with similar polyampholytic structures in their PDs mediating attractions) would disrupt fascicles, and impair neuronal polarity crucial to healthy neurons.

This highly unusual interaction between widely spaced surfaces is made possible by the non-uniform charge distribution of the PD of Tau, where segments of the NTT (which shift the active zone to the mid-layer) are followed by shorter, cationic/anionic domains enabling attraction between Tau on opposing microtubule surfaces. We hope this discovery will spur analytical and computer modelling efforts, which take into account the specific sequence of Tau, to more quantitatively describe the Tau-mediated interactions and, in particular, the effect on Tau-mediated microtubule attractions in the presence of Tau phosphorylation and disease-related hyperphosphorylation. Furthermore, generalizable principles derived from this system could serve as inspiration for polymer-directed assembling materials.

## Methods

### Purification of Tubulin and Tau

Tubulin was purified from MAP-rich microtubules extracted from bovine brains. MAP-rich microtubules were obtained from crude brain extract by three polymerization/depolymerization cycles, after which tubulin was separated from MAPs with a phosphocellulose anionic exchange column. Tubulin was suspended in PEM50 (50 mM PIPES (pH 6.8), 1 mM MgSO_4_ and 1 mM EGTA) with protein concentration between 7 and 12 mg ml^−1^, as measured by bovine serum albumin concentration standard. Solution was drop-frozen in liquid nitrogen and stored in a −70 °C freezer until use.

Tau was expressed in BL21(DE3) competent cells (Life Technologies, Carlsbad, CA) that were transfected with the pRK172 expression vector, coded for the appropriate WT isoform or truncated Tau. After incubation in auto-induction media (10 g of tryptone (CAS#: 91079–40–2), 5 g of yeast extract (CAS#: 8013–01–2), 0.5 g of dextrose (CAS#: 50–99–7), 2 g of α-D-lactose (CAS#: 5989–81–1) and 5 ml of glycerol (CAS#: 56–81–5) per litre of 25 mM Na_2_HPO_4_, 25 mM KH_2_PO_4_, 50 mM NH_4_Cl and 5 mM Na_2_SO_4_ in deionized (DI) water) for 24 h, cells were collected, lysed and resuspended in BRB80 buffer (80 mM PIPES at pH 6.8, 1 mM EGTA and 1 mM MgSO_4_). The solution was then bound to a phosphocellulose anionic exchange column, eluted with increasing concentration of (NH_4_)_2_SO_4_ in BRB80. Tau was further purified by a HiTrap hydrophobic interaction chromatography column (GE Healthcare Life Sciences, Pittsburgh, PA), eluted with decreasing concentration of (NH_4_)_2_SO_4_ in BRB80. Tau was then concentrated and buffer exchanged through successive centrifugation cycles using Amicon Ultra-15 Centrifugal Units with MWCO=10,000 (EMD Millipore, Darmstadt, Germany). The concentration of each Tau stock was determined by SDS–polyacrylamide gel electrophoresis comparison with a Tau mass standard (originally measured via amino-acid analysis).

Truncated Tau mutants were designed via the QuikChange Site-Directed Mutagenesis Kit (Agilent Technologies, Santa Clara, CA) with appropriate introduction/deletion of start and stop codons: 3RSΔC (truncation of the entire CTT, deleting residues 280–352 of 3RS), 3RΔ(N-) (truncation of the anionic component of the NTT, deleting residues 2–117 of 3RL) and 3RΔN (truncation of the entire NTT, deleting residues 2–255 of 3RL). Truncated Tau mutants were then expressed/purified, as above.

### Sample preparation

After thawing frozen tubulin and Tau stocks, samples were prepared on ice, mixing tubulin, GTP and Tau such that final concentrations were 5 mg ml^−1^, 2 mM and appropriate molar ratio of Tau to tubulin, respectively, in a final volume of 50 μl of PEM50 buffer. Samples were then polymerized in a 37 °C for 40 min. If necessary, sample was brought to appropriate KCl concentration.

### Osmotic pressure samples

A previous study^34^ measured the osmotic pressure (in Pa), *P*, of an aqueous solution of varying concentrations (cg ml^−1^), *wt%*, of poly(ethylene oxide) (*M*_W_=105,000 g mol^−1^) at 35 °C, which was taken as an reasonable approximation of the behaviour of PEO-100k at 37 °C, absent further data. Data were fit to a second-order polynomial (following the mathematical form of a virial expansion) to determine a formula to relate an arbitrary PEO-100k concentration to a corresponding osmotic pressure (*P*, in Pa):


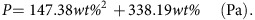


Alternatively, following a derivation in Rau *et al.*^35^, the osmotic pressure (*P*) on cylinders in a hexagonal lattice can be converted to a force per unit length between nearest cylinder pairs *f* as a function of the hexagonal lattice parameter *a*_H_:


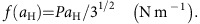


PEO-100k was used as the osmotic depletant of choice compared with better-characterized depletants to parameters unique to our system: as stable inter-microtubule distances of up to 41 nm were observed, the size of the depletant had to be equal or greater than that distance to create a concentration differential inside/outside the microtubule bundle. Prior work^36^ measured the radius of gyration (*R*_G_) of a function of PEO molecular weight (MW):


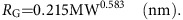


Thus, the effective depletant radius^21^, *a*=2*R*_G_*π*^−1/2^=19.95 nm, or an effective depletant diameter, *d*≈40 nm, satisfies our experimental conditions that polymer not penetrate the space between microtubules in microtubule bundles.

### Small-angle X-ray scattering

After polymerization, samples are loaded into 1.5-mm diameter quartz mark tubes (Hilgenberg GmbH, Malsfeld, Germany) and subsequently spun in a capillary rotor in a Universal 320R centrifuge (Hettich, Kirchlengern, Germany) at 9,500*g*, 37 °C for 30 min to protein density suitable for synchrotron SAXS. To ensure that structures were not induced by centrifugation, samples were observed over a period of 36 h, with no major changes to scattering or extracted parameters (Supplementary Fig. 1).

After centrifugation, varying concentration of PEO-100k in PEM50 was added for SAXS samples under osmotic pressure. Samples are subsequently sealed with epoxy and placed in a custom-made sample oven (maintained at 37 °C) with X-ray-transparent Kapton windows for scattering measurements.

SAXS measurements are carried out at the Stanford Synchrotron Radiation Laboratory (Palo Alto, CA) beamline 4–2 at 9 KeV (*λ*=1.3776 Å) with a Si(111) monochromator. Scattering data are taken with a 2D area detector (MarUSA, Evanston, Illinois) with a sample to detector distance of ≈3.5 m (calibrated with a silver behenate control). X-ray beam size on the sample was 150 μm in the vertical and 200 μm in the horizontal directions. To ensure reproducibility, scattering data were retaken for most samples at similar sample conditions using tubulin and Tau from different purifications and expressions/purifications, respectively.

### SAXS analysis

Scattering data were azimuthally averaged and small-angle scattering was subsequently background subtracted by fitting the minima of scattering intensities to a polynomial equation. Data were then fit to the appropriate model using a custom MATLAB fitting routine using the Levenberg–Marquadt non-linear fitting routine. Microtubules were modelled as homogenous, hollow cylinders (with no expected scattering from Tau/PEO due to low electron density relative to water) with ensemble-averaged inner radius <*r*_in_> (a fit parameter), wall thickness *δ* (49 Å, an input parameter^18^) and microtubule length *L* (20 μm, an input parameter for Tau-stabilized microtubules^16^), averaged all orientations in *q*-space:


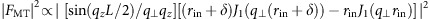


Where *q*_˙_ and *q*_*z*_ are wavevectors perpendicular and parallel to the tubular axis, and *J*_1_ is the Bessel function of order 1. The structure-factor peaks (at reciprocal lattice vector for a hexagonal array, |*G*_*hk*_|=*q*_10_(*h*^2^+*k*^2^+*hk*)^1/2^) were modelled as squared lorentzians with peak amplitude *A*_*hk*_ (a fit parameter) and peak width *κ*_*hk*_ (a fit parameter, with *κ*_10_ corresponding to the average bundle width *L*≈2(πln4)^1/2^/*κ*_*10*_)^13^:


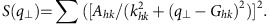


Fits of the intensity data, *I*(**q**)=|*F*_MT_|^2^ *S*(*q*_⊥_) yielded the hexagonal lattice parameter *a*_H_ (=4π/[3^1/2^*q*_10_]) and ensemble-averaged inner radius <*r*_in_>. *κ*_10_ was fit independently, while all other *κ*_*hk*_ fit simultaneously, with *κ*_*hk*_ approximately twice that of *κ*_10_.

### Plastic-embedded TEM sample preparation and TEM

Samples for thin sections were centrifuged to a pellet at 9,500*g* in 37 °C for 30 min. Supernatant was removed and pellet fixed with 2% glutaraldehyde and 4% tannic acid overnight. The pellet was stained with 0.8% OsO_4_ in PEM50 buffer for 1 h and subsequently rinsed four times with PEM50. Another stain of 1% uranyl acetate stain was applied for 1 h and rinsed with DI water.

Fixed and stained pellets were subsequently dehydrated with 25/50/75/100% solutions of acetone in DI water for 15 min apiece. Samples were embedded in resin, then embedded in spur plastic and incubated overnight, with resin poured into flat embedding moulds and held at 65 °C for 48 h and cooled overnight.

Plastic-embedded samples were then cut to ≈50-nm slices with a microtome (Ted Pella, Redding, CA) and transferred to highly stable Formvar carbon-coated copper EM grids (Ted Pella, Redding, CA). Data were taken using the JEOL 1230 Transmission Electron Microscope.

### DIC Samples and DIC

A SensiCam CCD camera (Cooke, Auburn Hills, MI) mounted on a Nikon Diaphot 300 with Xenon lamp (Sutter Instrument, Novato, CA) was used for optical microscopy measurements. Samples were centrifuged to a pellet at 9,500*g* for 30 min in 37 °C and placed between two microscopic slides sealed by wax. Images were taken while slides were kept at 37 °C by heat stage.

### Calculation of *R*_G_

Previously, the radius of gyration (*R*_G_) of WT Tau and truncated Tau domains in solution were found^20^,^37^ to scale as an unstructured protein with random-coil behaviour, with *R*_G_=0.1927*N*^0.588^ nm, which was subsequently used to calculate the *R*_G_ of the PD and truncated Tau used in our experiments.

### Data availability

The authors declare that the data supporting the findings of this study are available within the article, and its Supplementary Information files, or from the authors on reasonable request.

## Additional information

**How to cite this article**: Chung, P.J. *et al.* Tau mediates microtubule bundle architectures mimicking fascicles of microtubules found in the axon initial segment. *Nat. Commun.* 7:12278 doi: 10.1038/ncomms12278 (2016).

## Supplementary Material

Supplementary Information Supplementary Figure 1
