# Supplementary material for: Tau mediates microtubule bundle architectures mimicking fascicles of microtubules found in the axon initial segment
Source: Nat Commun. 2016 Jul 25;7:12278. doi: 10.1038/ncomms12278 (PMC4962469; doi:10.1038/ncomms12278)
Supplement: Supplementary Information — Supplementary Figure 1 [file ncomms12278-s1.pdf]

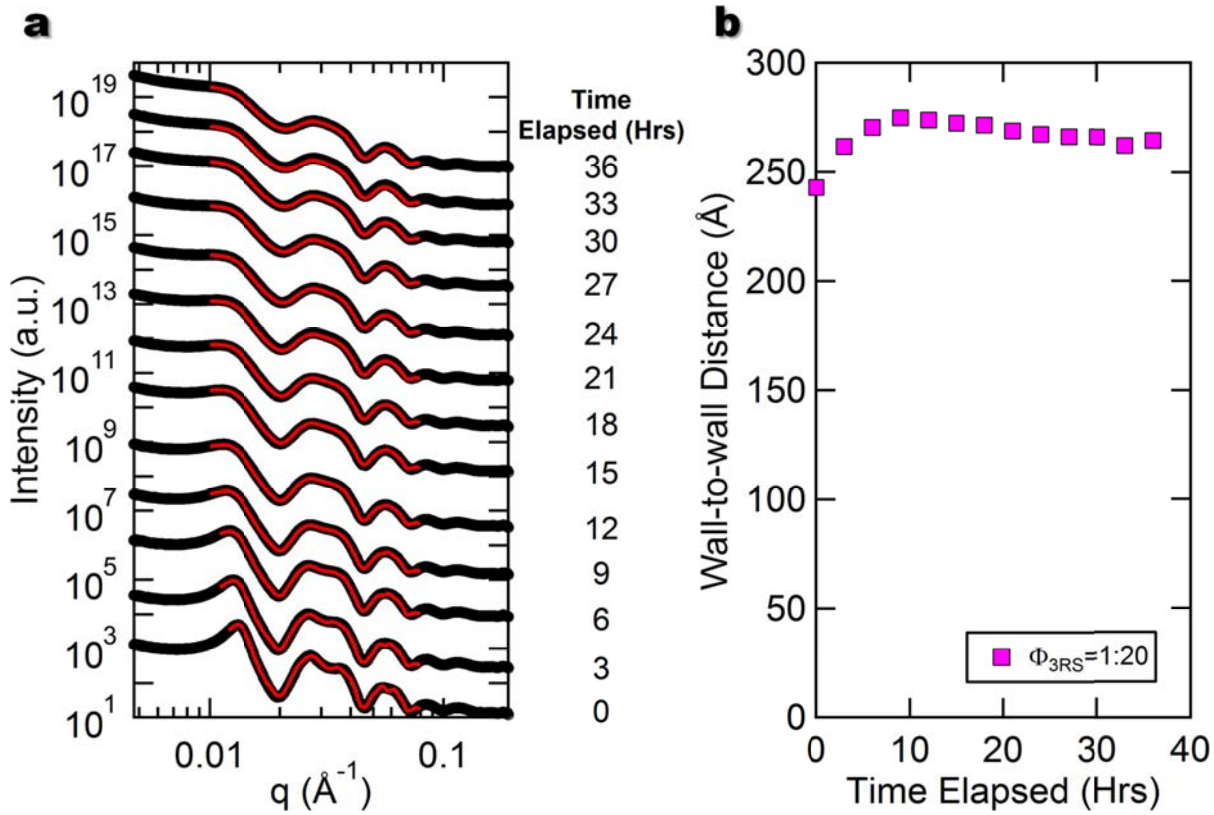

Supplementary Figure 1. Tau-mediated widely-spaced microtubule bundles are stable over time at 37˚ C in 2mM GTP. **a,** Despite being widely-spaced microtubules (over 30-40 times the electrostatic screening length), ongoing microtubule dynamic instability (no GTP regeneration), and being in solution at physiological temperature, scattering consistent with microtubules in hexagonal arrays is still apparent even after 36 hours. **b,** The corresponding wall-to-wall distances of bundles extracted from scattering data in (**a**) are stable over 36 hours after an initial increase over the initial 6-9 hours, indicating the likelihood widely-spaced bundles are a true energy minimum. Data presented in paper is of scattering at the 12-hour mark.
